# Continuing epidural analgesia during the second stage and ACOG definition of arrest of labor on maternal‐fetal outcomes

**DOI:** 10.1111/aas.13611

**Published:** 2020-05-14

**Authors:** ShengXing Zheng, Wenwen Zheng, Tianqi Zhu, Haiyan Lan, Qian Wang, Xiao Sun, MingPin Hu

**Affiliations:** ^1^ Department of Anesthesia The 2nd Affiliated Hospital and Yuying Children’s Hospital of Wenzhou Medical University Wenzhou Zhejiang People’s Republic of China; ^2^ Department of Anesthesia The First Affiliated Hospital of Wenzhou Medical University Zhejiang China

**Keywords:** Apgar scores, cesarean delivery, epidural analgesia in second stage of labor, NICU admissions

## Abstract

**Background:**

Despite an increase in the rates of epidural labor analgesia, continuation of epidural labor analgesia in the second stage of labor (CEADSSOL) was interrupted by care providers due to fears of increased risk of operative delivery and adverse neonatal outcomes. Therefore, we evaluated the effect of CEADSSOL and the newer American College of Obstetricians and Gynecologists (ACOG) definition of arrest of labor on the length of secondary stage of labor, newborn outcomes, and mode of delivery.

**Methods:**

This is a retrospective cohort study. Data collection began during March 2014 and ended in May 2015, 1 year after implementation of both interventions. The primary outcome was the length of secondary stage of labor, mode of delivery and neonatal outcome (Apgar < 7, at 5 minutes). The implementation of continuing epidural analgesia during the second stage of labor was performed with 0.08%‐0.15% ropivacaine and 0.1‐0.2 µg/mL sufentanil.

**Results:**

There were a total 10 414 deliveries during the study period. The length of the second stage of labor has no significant differences among groups. The cesarean delivery rate decreased 4.1% (36% vs 40.1%, *P* = .0038). Moreover, no significant difference was found in neonatal Apgar scores less than 7 at 5 minutes between two phases. Maternal outcomes remained unchanged. Post‐intervention neonatal parameters including NICU admissions (*P* < .001), incidences of antibiotics usage (*P* < .0001), intubation (*P* = .0003), and 7 days mortality (*P* = .0020) were remarkably reduced compared to pre‐interventions.

**Conclusion:**

The important finding of this study was the improvement in neonatal outcomes by implementing two simultaneous interventions without a cost of increased operative delivery.

**Editorial Comment**

Epidural labor analgesia should to be employed appropriately in different stages of labor. In this retrospective analysis of a large, recent, single‐center cohort which was focused on epidural analgesia during the second stage of labor, maternal and neonatal outcomes are presented. Epidural analgesia in the second stage of labor had some favorable associations to both in this cohort.

## 1 INTRODUCTION

The second stage of labor, as a most important part of the childbearing process, was under increased scrutiny due to the rapidly escalating use of cesarean delivery (CD).^1^ Epidural analgesia seems to be the most effective method of labor analgesia.^2^, ^3^ Our previous study demonstrated that utilization of epidural labor analgesia in the first stage of labor delivery reduced cesarean rate and was safe to both parturients and newborns.^4^ Despite an increase in the rates of epidural analgesia and improved maternal outcomes, continuing epidural analgesia during the second stage of labor (CEADSSOL) was interrupted by care providers due to fears of increased risk of operative vaginal, CD and adverse neonatal outcomes.^4^ Also, parturients and some obstetric care providers are quick to proceed to CD with arrest of labor with the belief that CD may be safer for the fetus.^5^

Several literature have reported that CEADSSOL was associated with prolonged duration of the second stage of labor and increased risk of instrumental vaginal delivery and CD,^6^, ^7^, ^8^, ^9^ which was not shown to be related to the adverse neonatal outcomes.^10^, ^11^ A randomized study reported that CEADSSOL witha low concentration of local anesthetic had no effect on the duration of the second stage of labor compared with a placebo infusion.^12^ Currently, there are no large‐scale studies focusing on newborn clinical outcomes and mode of delivery associated with CEADSSOL in China. Therefore, this study evaluated the implementation of CEADSSOL and the American College of Obstetricians and Gynecologists (ACOG) statement in 2014 on proceeding to CD following arrest of labor on neonatal outcomes and mode of delivery.^13^, ^14^ The primary aim of this study was to analyze the association between the pre‐ and post‐guideline implementationon on the length of secondary stage of labor, mode of delivery, and neonatal outcome (Apgar < 7, at 5 minutes).

## 2 MATERIALS AND METHODS

This retrospective study was approved by the institutional review board for clinical research from the Second Affiliated Hospital of Wenzhou medical university (Wenzhou, China). This study was conducted during the period March 2014‐May 2015, 1 year after implementation of both interventions (CEADSSOL and the ACOG statement in 2014^13^) using an electronic medical record system and verified using case logs in the labor and delivery (L & D) suites. All deliveries during the study period were included.

In pre‐both interventions period (pre‐phase), data collection for the present study occurred between March 2014 and May 2014. If there was no contraindication, epidural analgesia was provided to the parturients who requested for labor analgesia. The procedure of epidural analgesia was according to our previous study.^4^ Briefly, after epidural catheter placement at L2‐3 or L3‐4, the patient received the modified No Pain Labor N’Delivery (NPLD) protocol for epidural labor analgesia, consisting of an initial bolus of 8‐10 mL of 0.08%‐0.15% ropivacaine with sufentanil (0.1‐0.2 µg/mL) followed by an infusion of the same solution at 8‐10 mL/h with a patient‐controlled epidural analgesia pump. When the cervix was almost fully open, the analgesia administration was terminated by midwives for the second stage of labor.

In both interventions period (post‐phase), data collection for the present study began on June 2014 and continued until May 2015. During the first stage of labor, epidural analgesia was provided as in pre‐both interventions period. During the second stage, the modified NPLD protocol with epidural labor analgesia of 0.08%‐0.15% ropivacaine and sufentanil 0.1‐0.2 mcg/mL was used continuously from the first stage of labor, and the definitions regarding arrest of labor were adapted to the ACOG consensus clinically as well. In this period, the obstetric‐specific emergency response systems including timely resource recruitment and prevention of obstetric morbidity (such as fetal asphyxia) were established. Obstetric emergency team members (OBET) included the obstetricians, the midwives, the anesthesiologists, the charge and operating theatre nurses, and the neonatologists.

Decisions regarding terminating and changing participants’ epidural infusion, instrumental vaginal or operative deliveries were made by the obstetric care providers according to maternal or fetal indications. The main regimen used to treat breakthrough pain during the first or second stage of labor was 0.15% ropivacaine 5‐10 mL, with or without 5 µg sufentanil. Data were compared between pre‐phase (03/2014‐05/2014) and the post‐phase (06/2014‐05/2015) and between the earlier post‐phase (06/2014‐08/2014) and later post‐phase (03/2015‐05/2015).

The primary outcome was the length of secondary stage of labor, mode of delivery, and neonatal outcome (Apgar < 7, at 5 minutes). The secondary outcomes included use of episiotomy, perineal laceration, and other neonatal parameters such as NICU admissions, incidences of antibiotics usage, intubation, 7 days infant mortality, and neonatal Apgar scores. Apgar scores were assessed by midwives following routine vaginal deliveries and by operating room nurses following uncomplicated cesarean section. Apgar scores were determined by a neonatologist for high‐risk vaginal deliveries and by an anesthesiologist and/or neonatologist for high‐risk CD.

### 2.1 Statistical analysis

The primary outcome (the length of secondary stage of labor, mode of delivery, and neonatal outcome [Apgar < 7, at 5 minutes]) was compared using the χ^2^ test and *t* test. *P* < .05 is chosen for rejecting the null hypotheses. The secondary outcomes included the following: Continuous outcomes were analyzed using unpaired *t*‐test or Mann‐Whitney *U* test when not normally distributed. The Shapiro‐Wilk test was used to assess the distribution of the data. Categorical data were analyzed using the χ^2^ test or Fisher exact test. Differences in the outcome rates and 99% confidence intervals (CIs) are reported. The analyses were performed with Stata Statistical Software 15.0 (StataCorpLLC, TX, USA).

## 3 RESULTS

**Figure 1:**
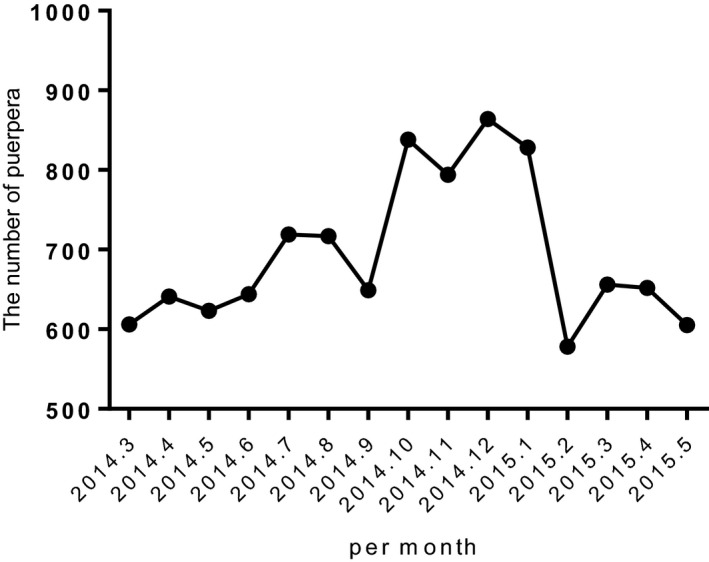
Number of deliveries per month

There were a total 10 414 deliveries during the study period with 1870 parturients in the pre‐intervention phase (pre‐phase) and 8544 cases in the post‐intervention phase (post‐phase). The monthly deliveries increased from 623 in pre‐phase period to 712 in post‐phase period (Figure 1). The demographic data about maternal age, maternal weight, height, body mass index, and birth weight did not show significant differences between pre‐phase and post‐phase (*P* > .05) (Table 1).

**Table 1:** Demographic data in each group. Data reported as mean ± SD

Group	Maternal age (y)	Maternal height (cm)	Maternal weight (kg)	Body mass index	Birth weight (g)
Pre‐intervention phase	26.77 ± 0.51	160.47 ± 1.39	70.57 ± 1.19	27.43 ± 0.92	3267 ± 36.51
Post‐intervention phase	26.3 ± 0.28	160.38 ± 0.57	69.92 ± 0 0.73	27.19 ± 0.28	3301.17 ± 33.82

The primary outcome, the length of the second stage of labor, has no significant differences among groups (Table 2). The CD rate decreased by 4.1% (36% vs 40.1%, *P* = .0038) (Table 2), while intrapartum CD (*P* = .4796) (Table 2) did not show significant differences between pre‐phase and post‐phase. Moreover, no significant difference was found in neonatal Apgar scores <7 at 5 minutes for vaginal and cesarean deliveries between pre‐intervention phase and post‐intervention phase (Table 2).

**Table 2:** Impact of continuing epidural analgesia during the second stage of labor and the newer ACOG definition of arrest of labor on the length of secondary stage of labor, mode of delivery and neonatal outcome. Data reported as mean (99% CI) and N (%)

Clinical outcomes	Pre‐phase	Post‐phase	Difference *P*	Earlier post‐phase	Later post‐phase	Difference *P*
Monthly deliveries	623 (523, 724)	712 (625, 799)	89 (14.3%), *P* = .0748	693 (448, 938)	638 (475, 800)	−56 (−8.1%), *P* = .0667
Epidural analgesia (%)	43.6 (32.4, 54.8)	57.2 (52.7, 61.6)	13.6%, *P* = .0003	50.6 (34.8, 66.3)	61.2 (35.8, 86.7)	10.6%, *P* = .0121
The length of secondary stage of labor	56.9 (27.5, 86.3)	54.9 (52.1, 57.7)	−1.96%, *P* = .7969	53.6 (50.6, 56.6)	53.8 (30.1, 77.4)	0.17%, *P* = .526
Maternal rates (%)						
Cesarean delivery (%)	40.1 (29.1, 51.2)	36.0 (34.2, 37.8)	−4.1%, *P* = .0038	37.8 (28.8, 46.9)	35.9 (32.4, 39.5)	−1.9%, *P* = .0615
Intrapartum cesarean deliveries (%)	6.03 (4.5, 7.6)	6.06 (5.3, 6.8)	0.03%, *P* = .4796	6.3 (3.7, 8.9)	5.6 (4.5, 6.7)	−0.7%, *P* = .0339
Episiotomy and perineal laceration (%)	11.6 (−3.3, 26.5)	12.0 (10.2, 13.8)	0.4%, *P* = .3839	9.5 (−5.3, 24.3)	12.7 (6.3, 19.1)	3.2%, *P* = .0597
Forceps (%)	1.3 (−3.5, 6.1)	1.6 (1.0, 2.1)	0.3%, *P* = .264	1.09 (−2.5, 4.6)	1.14 (−2.5, 4.6)	0.05%, *P* = .4605
Neonatal outcomes (%)						
Vaginal deliveries Apgar score ≤ 7 at 5 min (%)	0.37 (−1.03,1.77)	0.18 (−0.0.01, 0.37)	−0.19%, *P* = .0964	0.14 (−1.24,1.52)	0.31 (−1.44, 2.06)	0.17%, *P* = .2410
Cesarean deliveries Apgar score ≤ 7 at 5 min (%)	0.32 (−1.27, 1.92)	0.18 (−0.02, 0.38)	−0.14%, *P* = .1863	0.14 (−0.66, 0.94)	0.31 (−1.44, 2.07)	0.17%, *P* = .2094

The neonatal outcomes between pre‐intervention phase and post‐intervention phase were comparable, and post‐intervention NICU admissions, were significantly reduced by 8.1% (14.1% vs 5.9%, *P* < .001) compared to pre‐interventions (Table 3). There was also no significant difference in length of NICU stay between two groups. Post‐intervention neonatal parameters including incidences of antibiotics usage (*P* < .0001), intubation (*P* = .0003), and 7‐day mortality (*P* = .0020) were remarkably reduced compared to pre‐interventions (Table 3). There was no significant differences in newborn outcomes between the first three and the last 3 months post‐interventions. (Tables 2 and 3). Maternal outcomes including monthly rates of forceps (*P* = .264), episiotomy and perineal laceration (*P* = .3839), and intrapartum CD (*P* = .4796) were not statistically different between pre‐intervention phase and post‐intervention phase (Table 2). The epidural analgesia rate increased by 13.6% in post‐intervention phase (57.2% vs 43.6%, *P* = .0003) compared with pre‐intervention phase (Table 2). There was no maternal death during the study period.

**Table 3:** Impact of continuing epidural analgesia during the second stage of labor and the newer ACOG definition of arrest of labor on newborn outcomes. Data reported as mean (99% CI) and N (%)

Clinical outcomes	Pre‐phase (99% CI)	Post‐phase (99% CI)	Difference *P*	Earlier post‐phase (99% CI)	Later post‐phase (99% CI)	Difference *P*
Overall NICU admission (%)	14.1 (7.3, 21.0)	5.9 (4.7, 7.1)	−8.1%, *P* < .0001	6.8 (3.5, 10.2)	6.5 (2.4, 10.7)	−0.3%, *P* = .2907
Overall NICU length (days)	15.6 (−2.4, 33.5)	14.8 (13.1, 16.5)	−0.8, *P* = .2863	14.5 (13.3, 15.7)	15.6 (1.4, 29.8)	1.1, *P* = .2440
Overall intubation (%)	2.2 (−0.3, 4.6)	1.1 (0.8, 1.4)	−1.1%, *P* = .0003	1.05 (−0.8, 2.9)	1.3 (0.6, 2.0)	0.25%, *P* = .1321
Antibiotics usage (%)	7.3 (1.9, 12.6)	3.4 (2.6, 4.3)	−3.9%, *P* < .0001	4.6 (−1.3, 10.5)	3.4 (2.3, 4.6)	−1.1%, *P* = .0696
7‐day mortality	0.63 (−1.68, 2.95)	0.16 (0.03, 0.30)	−0.47%, *P* = .0020	0.19 (−1.04, 1.41)	0.05 (−0.46, 0.56)	−0.14%, *P* = .1846
Vaginal deliveries						
NICU admission (%)	7.8 (4.2, 11.3)	3.2 (2.6, 3.9)	−4.5%, *P* < .0001	3.7 (2.1, 5.3)	3.9 (2.4, 5.4)	0.3%, *P* = .1567
NICU length (days)	15.5 (−8.8, 39.4)	14.6 (11.6, 17.5)	−0.9, *P* = .3350	11.9 (4.7, 19.1)	16.9 (4.3, 29.6)	5.0, *P* = .0131
Average 1‐min Apgar score	8.4 (7.7, 9.1)	8.5 (8.1, 8.9)	0.1, *P* = .3011	8.7 (4.1, 13.3)	8.8 (6.7, 10.8)	0.1, *P* = .4480
Average 5‐min Apgar score	9.38 (7.87, 10.88)	9.44 (9.21, 9.67)	0.06, *P* = .4480	9.61 (7.23, 11.99)	9.42 (7.87, 10.97)	−0.19, *P* = .2782
Intubation (%)	1.06 (−0.67, 2.80)	0.59 (0.32, 0.85)	−0.47%, *P* = .0131	0.53 (−1.51, 2.58)	0.57 (−0.7., 1.84)	0.04%, *P* = .4435
Cesarean deliveries						
NICU admission (%)	6.4 (3.0, 9.7)	2.7 (1.9, 3.4)	−3.7%, *P* < .0001	3.2 (1.2, 5.1)	2.6 (−0.6, 5.8)	−0.6%, *P* = .1003
NICU length (days)	15.7 (1.5, 29.8)	15.0 (13.1, 17.0)	−0.7, *P* = .3341	17.1 (9.2, 25.0)	14.3 (−1.6, 30.1)	−2.8, *P* = .0925
Average 1‐min Apgar score	8.1 (6.2, 10.0)	8.3 (8.1, 8.6)	0.2, *P* = .1316	8.3 (7.5, 9.1)	8.31 (7.0, 9.6)	0.01, *P* = .4669
Average 5‐min Apgar score	9.36 (7.07, 11.65)	9.35 (9.07, 9.63)	−0.01, *P* = .4892	9.46 (8.12, 10.80)	9.24 (8.20, 10.28)	−0.22, *P* = .1307
Intubation (%)	1.12 (0.38, 1.86)	0.52 (0.27, 0.76)	−0.60%, *P* = .0015	0.52 (−1.44, 2.47)	0.73 (0.11, 1.36)	0.21%, *P* = .1757

Moreover, the scores of satisfaction of parturients were investigated by a survey. About 1500 questionnaires were issued and 1205 were collected from March 2014 to December, 2014. The scores of satisfaction in second stage analgesia in pre‐intervention group (82 ± 7) were lower than the post‐ intervention group (91 ± 5) (*P* < .05) (Table 4).

**Table 4:** Scores of satisfaction of parturients in each group. Data reported as mean ± SD, #*P* < .01 compared with post‐phase group (0 = dissatisfaction, 100 = great satisfaction)

Group	Scores of satisfaction in the first stage analgesia	Scores of satisfaction in the second stage analgesia
Pre‐phase (326)	95 ± 6	82 ± 7^#^
Post‐phase (879)	94 ± 7	91 ± 5

## 4 DISCUSSION

In our study, we demonstrated that neonatal outcomes were improved by implementing two simultaneous interventions (CEADSSOL and the ACOG statement in 2014) without a cost of increased operative delivery. In fact, the CD rate decreased in post‐intervention phase compared to pre‐intervention phase. There was not a statistical association between two simultaneous interventions and a longer lasting second stage. Furthermore, we found that monthly forceps rates, incidence of episiotomy and perineal laceration, and intrapartum CD remained unchanged between two phases.

The epidural analgesia is well used in the labor and delivery floor in China from 2010. We previously reported that the epidural analgesia used during the first stage of the labor did not affect maternal and neonatal outcomes.^4^ However, some Chinese anesthesiologists and obstetric providers discontinue epidural analgesia during the second stage of labor. There is wide variation from center to center in epidural rates occurring in 46% of deliveries (range 14%‐85%) and 89% of deliveries discontinued the epidural analgesia in the second stage at the hospital where this study was performed.^5^ The potential effect of epidural analgesia on the second stage of labor remains debatable. The studies showed that women who received CEADSSOL had a longer second stage of labor, increased use of oxytocin augmentation, and an increased risk of instrumental vaginal delivery.^15^, ^16^, ^17^ In addition, a meta‐analysis that was performed analyzing the possible consequences of discontinuing epidural analgesia during the second stage of labor,^18^ which only found there was an significant increase in pain. Quite many parturients require CD because labor pain worriness. Also, cesarean section was believed to be lifesaving for the fetus or/and the parturients. However, over the last decades, CD rate increased rapidly, which has not been able to further lower the perinatal mortality.^19^, ^20^, ^21^

Our study found that the labor epidural analgesia usage went up, while the CD rate decreased in post‐intervention phase. With better ability for and interpretation of intrapartum fetal monitoring,^22^, ^23^ we also encouraged women with previous cesarean deliveries to attempt vaginal delivery. Furthermore,our study demonstrated that there was no significant change in the incidence of episiotomy and perineal laceration, the intrapartum CD rate, and monthly forceps rates between both modes, which was consistent with previous studies.^16^, ^24^, ^25^ Due to the obstetric‐specific emergency response systems, there was no maternal death during the study period.

A longer duration of the second stage labor is related to increased risks of both maternal and perinataladverse outcomes,^10^ which may depend on the intervention of obstetric care providers.^26^ The newer ACOG definition of arrest of labor with the use of epidural analgesia suggest that the duration of the second stage labor could be at least 3 hours of pushing in multiparous women and 4 hours of pushing in nulliparous women before diagnosing arrest of labor. Of note, a higher epidural local anesthetic concentration is associated with more deleterious effects on the second stage of labor.^27^ Although ropivacaine and bupivacaine provided equi‐effective analgesia,^28^ ropivacaine produced less motor block than bupivacaine.^29^ In our study, we used epidural labor analgesia with 0.08%‐0.15% concentrations of ropivacaine and sufentanil 0.1‐0.2 mcg/mL, which contribute to less motor block but clinically equal potent.^30^ We found, as for the length of the second stage of labor, there were nostatistical changes in both modes.

In the present study, a reduced rate of NICU admissions was noticed in the woman who received CEADSSOL, and we also found no significant difference inneonatal Apgar scores <7 at 5 minutes for vaginal and cesarean deliveries between pre‐intervention phase and post‐intervention phase,which was consistent with the previous study.^12^ We also revealed that neonatal outcomes including incidences of antibiotics usage, intubation, and 7‐day mortality were associated positively by implementing two simultaneous interventions. There was also no significant difference in the length of NICU stay. Other large cohort studies also suggest that women who receive CEADSSOL achieved delivery without adverse neonatal outcomes.^15^ These results, taken together, indicating utilization of two simultaneous interventions (CEADSSOL and the ACOG statement in 2014), could change obstetric practice with improvements in neonatal outcomes and delivery outcomes.

Antenatally, 90% of all parturients anticipated a need for pain relief during labor.^31^ Over 80% of all parturients describedt heir pain as very severe to intolerable in the delivery.^31^ Therefore, it may be as important to assess satisfaction with labor care and the effectiveness of labor analgesia. We found that the scores of satisfaction in second stage analgesia were higher after implementing two simultaneous interventions. The previous study also demonstrated that withdrawal of epidural analgesia resulted in lower satisfaction scores.^12^

The current study has some limitations needed to be addressed. First, the causality between the implementation of two simultaneous interventions and the length of second stage of labor; increased risk of instrumental vaginal delivery and CD cannot be established in our study due to limited to available data. Second,the data were obtained from one single institution, and thus may not be generalizable to populations in other institutes with different labor analgesia management. We analyzed data from parturients who were only Asians**.** Therefore, the study from other ethnicities would be required to compare with our findings due to possible existence of regional, cultural,or ethnic differences. Third, this retrospective character was neither able to control the groups before and after nor made sure that intervention was carried out properly. Finally, recall bias would affect the association between the exposure and outcomes. For example, blood loss and umbilical artery pH were missing. To determine recall bias, a further prospective study will be designed.

## 5 CONCLUSION

The important finding of this study was the improvement in neonatal outcomes by implementing two simultaneous interventions without a cost of increased operative delivery. In fact, the simultaneous interventions are associated with decreased CD rates in the post‐intervention period with sustained effects. Therefore, based on the findings in our study, there was no evidence of worse neonatal or maternal outcomes.

## CONFLICT OF INTEREST

The authors declare that they have no conflict of interest.

## AUTHOR CONTRIBUTIONS

SXZ: data collection or management, data analysis,and manuscript writing/editing. HYL: data collection or management. QW: data collection or management and data analysis. QQL: protocol/project development. TQZ: data analysis. XS: data analysis. MPH: protocol/project development.

## ETHICAL APPROVAL

All procedures performed in studies involving human participants were in accordance with the ethical standards of the institutional and/or national research committee and with the 1964 Helsinki declaration and its later amendments or comparable ethical standards.
